# P-149. Clinical Profile and Antimicrobial Resistance Patterns of Salmonella Infection in Guatemala City

**DOI:** 10.1093/ofid/ofae631.354

**Published:** 2025-01-29

**Authors:** Ana Elisa Rosales, Andrea Barrios, Jarleny Orozco, Laura Valenzuela, Luis Rodriguez, Diego Erdmenger

**Affiliations:** Hospital General San Juan de Dios, Guatemala, Alta Verapaz, Guatemala; Hospital General San Juan de Dios, Guatemala, Alta Verapaz, Guatemala; Hospital General San Juan de Dios, Guatemala, Alta Verapaz, Guatemala; Hospital General San Juan de Dios, Guatemala, Alta Verapaz, Guatemala; Hospital General San Juan de Dios, Guatemala, Alta Verapaz, Guatemala; Hospital General San Juan de Dios, Guatemala, Alta Verapaz, Guatemala

## Abstract

**Background:**

Salmonella infection is the second most common food-borne disease worldwide, causing over 155,000 deaths per year. Infection rates in low- and middle-income countries surpass those of high-income countries.

Several risk factors have been associated with higher mortality rates due to Salmonella infection, such as age, HIV infection, and antimicrobial resistance.

Although frequent in Central America, few data on risk factors associated to invasive Salmonellosis and mortality are available in the region.

Population General Characteristics

**Figure:**
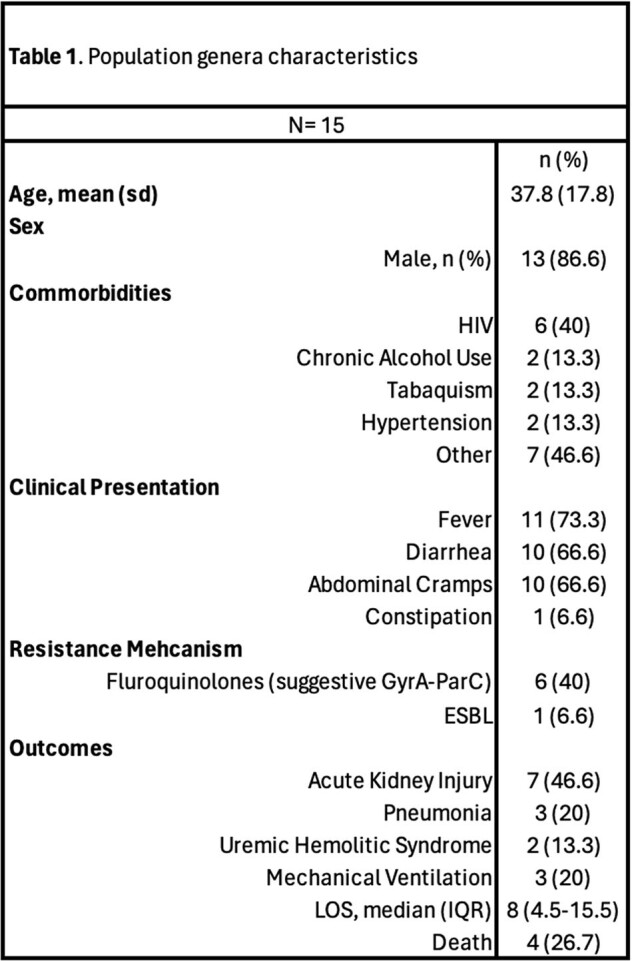

**Methods:**

A retrospective cohort study was conducted at a third-level reference hospital in Guatemala City from 2022 to 2023. All cases of Salmonella infection identified through blood and stool cultures were included and evaluated for clinical factors and interest outcomes. Antimicrobial resistance patterns were identified according to the Clinical and Laboratory Standard Institute (CLSI) guidelines.

Clinical characteristics associated with mortality

**Figure:**
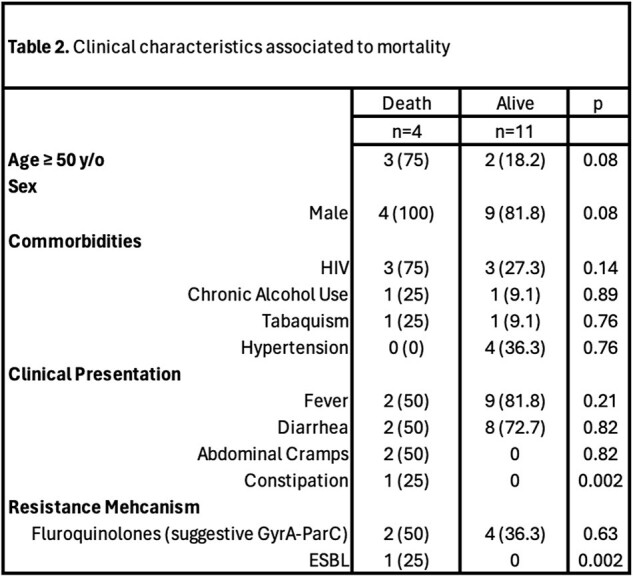

**Results:**

A total of 15 confirmed cases of Salmonella infection were identified. The mean age of the patients was 37.8 years (SD = 17.8). The majority of were male (86.6%). Over 73% had at least one comorbidity, with HIV being the most frequent, followed by chronic alcohol consumption.

The most common presentation included fever, diarrhea, and abdominal cramps. Only 6.6% of patients reported constipation. Over 46% of strains exhibited at least one mechanism of resistance, with 40% of showing resistance to fluoroquinolones, suggesting the presence of a GyrA-ParC phenotype.

The most common complication identified was Acute Kidney Injury in 46.6% of cases, with Hemolytic Uremic Syndrome present in 13.3% of cases. The overall mortality rate due to salmonellosis was 26.6% (Table 1).

Age over 50 years, sex, HIV infection, and ESBL resistance were significantly associated with in-hospital mortality, after adjusting for other clinical variables (Table 3).

Adjusted variables associated with mortality

**Figure:**
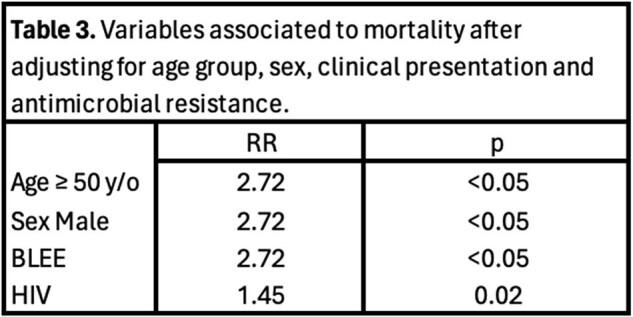

**Conclusion:**

The study identified a higher resistance to fluoroquinolones and case-mortality compared to other reports in the region. Although ESBL-producing strains were rare in the study, this resistance pattern was significantly associated with mortality due to salmonella infection. The uncontrolled use of antibiotics in the country may present as a major concern in the future treatment and management of salmonellosis.

**Disclosures:**

**All Authors**: No reported disclosures

